# Timing of Malaria Infection during Pregnancy Has Characteristic Maternal, Infant and Placental Outcomes

**DOI:** 10.1371/journal.pone.0074643

**Published:** 2013-09-18

**Authors:** Linda Kalilani-Phiri, Phillip C. Thesing, Osward M. Nyirenda, Patricia Mawindo, Mwayi Madanitsa, Gladys Membe, Blair Wylie, Abbey Masonbrink, Kingsley Makwakwa, Steve Kamiza, Atis Muehlenbachs, Terrie E. Taylor, Miriam K. Laufer

**Affiliations:** 1 Department of Community Heath, University of Malawi College of Medicine, Blantyre, Malawi; 2 Blantyre Malaria Project, University of Malawi College of Medicine, Blantyre, Malawi; 3 Department of Obstetrics and Gynaecology, University of Malawi College of Medicine, Blantyre, Malawi; 4 Department of Obstetrics and Gynecology, Massachusetts General Hospital, Boston, Massachusetts, United States of America; 5 Department of Pediatrics, University of Maryland School of Medicine, Baltimore, Maryland, United States of America; 6 Department of Histopathology, University of Malawi College of Medicine, Blantyre, Malawi; 7 Department of Pathology, University of Washington School of Medicine, Seattle, Washington, United States of America; 8 Department of Internal Medicine, College of Osteopathic Medicine, Michigan State University, E. Lansing, Michigan, United States of America; 9 Center for Vaccine Development/HHMI, University of Maryland School of Medicine, Baltimore, Maryland, United States of America; The George Washington University Medical Center, United States of America

## Abstract

We conducted a clinical study of pregnant women in Blantyre, Malawi to determine the effect of the timing of malaria infection during pregnancy on maternal, infant and placental outcomes. Women were enrolled in their first or second trimester of their first or second pregnancy and followed every four weeks until delivery. Three doses of sulfadoxine-pyrimethamine were given for intermittent preventive treatment for malaria, and all episodes of parasitemia were treated according to the national guidelines. Placentas were collected at delivery and examined for malaria parasites and pigment by histology. Pregnant women had 0.6 episodes of malaria per person year of follow up. Almost all episodes of malaria were detected at enrollment and malaria infection during the follow up period was rare. Malaria and anemia at the first antenatal visit were independently associated with an increased risk of placental malaria detected at delivery. When all episodes of malaria were treated with effective antimalarial medication, only peripheral malaria infection at the time of delivery was associated with adverse maternal and infant outcomes. One quarter of the analyzed placentas had evidence of malaria infection. Placental histology was 78% sensitive and 89% specific for peripheral malaria infection during pregnancy. This study suggests that in this setting of high antifolate drug resistance, three doses of sulfadoxine-pyrimethamine maintain some efficacy in suppressing microscopically detectable parasitemia, although placental infection remains frequent. Even in this urban setting, a large proportion of women have malaria infection at the time of their first antenatal care visit. Interventions to control malaria early and aggressive case detection are required to limit the detrimental effects of pregnancy-associated malaria.

## Introduction

Pregnancy is a unique period of vulnerability to malaria infection. This increased susceptibility is attributed to the ability of infected erythrocytes to sequester in the developing placenta, causing chronic infection and placental inflammation. In malaria-endemic areas, pregnancy-associated malaria causes maternal anemia and low infant birth weight [1]. Because malaria infection during pregnancy is often asymptomatic, the most common control strategy is the administration of sulfadoxine-pyrimethamine (SP) at regular intervals during the second and third trimesters irrespective of malaria diagnosis or clinical evidence of infection. This intermittent preventive treatment during pregnancy (IPTp) is designed to clear any malaria infection present at the time of treatment and also to provide post-treatment prophylaxis to prevent infection for a period of weeks. However, there is increasing concern that with widespread SP resistance throughout Africa, a new intervention to prevent pregnancy-associated malaria will need to be implemented [2]. Some alternatives that have been proposed and are currently being evaluated include more frequent IPTp with SP, replacement of SP with a more effective medication and also the replacement of IPTp with intermittent screening and treatment. Studies designed to evaluate new interventions generally enroll pregnant women when they present for their first antenatal care visit, often late in the second or even in the third trimester, do not include active malaria surveillance during the follow up period, and use placental malaria infection as the primary outcome. These studies are based on several assumptions: that interventions which begin at first antenatal visit are likely to impact outcomes, that episodes of peripheral malaria at all stages of pregnancy will lead to placental infection, and that placental malaria infection is associated with adverse outcomes.

We hypothesized that the detrimental effects of pregnancy-associated malaria would not be distributed uniformly throughout pregnancy and that the timing of the malaria infection would significantly affect placental pathology as well as clinical outcomes in mothers and infants. We designed a prospective study to examine the effect of timing of malaria infection on placental, maternal and infant outcomes. Within the context of the standard administration of SP IPTp, we enrolled women as early as possible during pregnancy and conducted intensive evaluation of maternal infection status as well as infant and maternal outcomes. We actively screened for malaria infection to determine the rates of microscopically detectable parasitemia during pregnancy and used a combination of histopathology and molecular techniques to detect malaria parasites in the placenta.

## Materials and Methods

### Study Site

The study was conducted in Ndirande, a peri-urban township of Blantyre in the southern region of Malawi. Malaria transmission occurs year-round, peaking in the rainy season from December to March. The government health center and antenatal clinic are the only public providers of health care for the catchment area with a population of over 200,000. Participants were recruited from the antenatal clinic. A maternity ward is also located on the health center compound, where deliveries attended to by nurse midwives occur 24 hours a day. Complicated deliveries are referred for obstetric care to Queen Elizabeth Central Hospital, the government referral hospital in southern Malawi, located approximately five kilometers from the health center.

### Study Population and Enrollment Procedures

All pregnant women residing within the health facility catchment area who were in the first or second pregnancy, at least 15 years old, and less than or equal to 28 weeks gestation were eligible for the study. Women with any major illnesses or who were on chronic antibiotic treatment were excluded from the study. Women known to be HIV-infected were initiated on trimethoprim-sulfamethoxazole prophylaxis according to the national policy, and thus not eligible to receive IPTp with SP; these women were excluded from the study. Informed consent was obtained before conducting any study procedures. On enrollment, study participants were assigned a study identification number. Using a standardized questionnaire, information was collected on demographic and socio-economic characteristics, and past obstetric and medical history. Detailed contact information was also collected and used to trace participants if they did not attend a scheduled visit. Clinical examination included blood pressure, axillary temperature and anthropometric measurements. Assessment of gestational age at enrollment was done using the last menstrual period and measurement of fundal height. A finger prick blood sample was obtained for preparation of blood smears, hemoglobin measurement (HemoCue AB) and storage of a dried blood spot on filter paper. All pregnant women received an insecticide-treated bed net and iron and folic acid supplementation according to standard procedures in the antenatal clinic. The first dose of SP IPTp was given at the enrollment visit or at 20 weeks gestation if enrollment occurred earlier in pregnancy.

### Antenatal Follow-Up

Study participants were seen at the clinic every four weeks and were encouraged to return every time they were ill. At every routine visit and any visit for an illness suspicious of malaria, a finger prick blood sample was collected to measure hemoglobin levels, to prepare blood smears, and to prepare a filter paper specimen. Women received SP as IPTp up to three times during their pregnancy, separated by at least four weeks. All episodes of malaria parasitemia detected at routine and sick visits were treated with an antimalarial drug in accordance with the national guidelines (quinine in the first trimester and artemether-lumefantrine in the second and third trimesters), regardless of symptoms. Women with evidence of severe malaria were referred to the hospital for evaluation and treatment. Parasitemia occurring within 28 days of the initial infection was considered to be the same episode.

### Delivery Procedures

Study participants were encouraged to deliver at the maternity ward at Ndirande Health Centre and those with complications were referred to Queen Elizabeth Central Hospital. After delivery, the placenta was stored and transported to the research clinic where placental blood was collected for parasite DNA detection. Biopsy specimens were preserved in formalin for histopathological processing. Newborns were assessed and gestational age was estimated using a modified Ballard score [3]. Infants were followed until they reached 14 weeks of age.

### Definitions

Women were classified as having anemia if they had a hemoglobin concentration less than 11 g/dL. Anemia was further classified as mild (9.0-10.9 g/dL), moderate (7.0-8.9 g/dL) or severe (<7.0 g/dL). Fever was defined as axillary temperature greater than or equal to 37.5 °C. A birth weight of less than 2500 grams was classified as low birth weight. Gestational age at enrollment was calculated based on the last menstrual period or by the fundal height if the last menstrual period was not known. Gestational age at birth was determined by combining information from the last menstrual period and the Ballard exam. If the gestational age by last menstrual period and the Ballard exam were less than two weeks apart, menstrual dating was considered the best obstetric estimate of gestational age. If there was a discrepancy of more than two weeks, fundal height was included in the determination of the best estimate and was used for evaluating birth outcomes. The first trimester was defined as conception through 13 weeks and the second trimester was from 14 through 27 weeks. A gestational age less than 37 weeks at delivery was classified as premature delivery. Birth weight for gestational age, based on the WHO growth curves less than -2 Z-score was considered small for gestational age [4].

### Laboratory procedures

#### Malaria diagnosis

Peripheral blood slides were Field stained and examined microscopically using a 100x oil immersion objective to detect and quantify parasitemia. A diagnosis of microscopically-detectable malaria infection was made when asexual stage malaria parasites were detected on a thick film. All slides were read by two microscopists and in cases of disagreement between the readings, the conflict was adjudicated by a third expert reader.

#### Placental biopsies

Placental biopsies were preserved in 10% neutral buffered formalin, embedded in paraffin wax, cut into four micron thick sections, and then stained with hematoxylin and eosin. Slides were examined for presence of malaria parasites and pigment. If parasites were seen on the slide, a placental blood specimen was used to extract and test for the presence of malaria DNA based on quantitative PCR, as described on our website (http://medschool.umaryland.edu/malaria/protocols.asp). Only specimens with parasites detected by histology and confirmed by PCR were considered positive for parasites. Biopsies with parasites or pigment were classified as placental malaria infection.

### Ethical Considerations

The study protocol and informed consent document was approved by the University of Malawi College of Medicine Research and Ethics Committee and the University of Maryland and Massachusetts General Hospital Institutional Review Boards. Written informed consent was obtained from all participants before conducting any study related activities. All participants were adults or mature minors and this determination was approved by the institutional review boards. The study consent included permission to assess the newborn infant at birth and through the first 14 weeks of life. Participants had the option to withdraw from the study at any time. All data were recorded and analyzed anonymously. Approval was also obtained from the Blantyre District Health Officer in charge of Ndirande Health Centre, the Blantyre City Assembly which is responsible for the Antenatal Clinic, and the head of the Obstetrics and Gynecology department at Queen Elizabeth Central Hospital and the University of Malawi College of Medicine.

### Statistical Analysis

Data analysis was performed using STATA version 10.0 software (Stata Corp., College Station, TX, USA). Demographic characteristics were described using frequencies and percentages for categorical data and means and 95% confidence intervals (CI) for continuous variables. All data collected from participants between enrollment and delivery or termination was included in the analysis. Episodes of malaria during the follow up period were recorded for participants who had at least one visit beyond the enrollment period. Time under observation began at enrollment and continued until delivery or the last visit prior to study termination, and individuals were considered continuously at risk while under surveillance. Proportions were compared using Fisher’s exact test and odd ratios were calculated using logistic regression. For the analysis of the association between the timing of malaria infection and outcomes, logistic regression was used to compare individuals who experienced malaria infection to individuals who did not experience malaria infection in each trimester. Infections in the second and third trimester were included in the model. Multivariate models were developed when more than one risk factor was associated with the dependent variable of interest. Independent variables were only included in the final model if they had an association with the dependent variable in the univariate analysis with a p-value equal to or less than 0.1.

## Results

### Baseline characteristics of study participants

A total of 450 women were enrolled into the study from June 2009 through June 2010. The median age of the participants was 20.1 years (95% CI 19.8-20.4). The majority of women were in their first pregnancy, had received some formal education and had adequate shelter and access to clean water. Approximately half of the women had slept under a bed net on the night before enrollment. The baseline characteristics of the pregnant women at enrollment are listed in Table 1. Eight percent (36/450) of women enrolled in their first trimester and the remaining women were in their second trimester. One hundred fifty-seven person years of follow up during pregnancy were accrued. The most common reasons for early study termination were migration outside the Blantyre area (n=71) and withdrawal of consent, usually due to the influence of a key family member (n=55).

**Table 1:** Characteristics of study participants at enrollment.

**Characteristic**	**Result Mean (95% confidence interval**)** or Number (%**)
Mean age, years	20.1 (19.8-20.4)
Education: None	1 (0.2)
Education: Primary school education	140 (31.1%)
Education: Secondary school education	303 (67.3%)
Education: Beyond secondary school	6 (1.3%)
Married	357 (82.3%)
Mean household size, number of people	3.4 (3.3-3.6)
Access to piped water	445 (98.9%)
Mud or brick walled house	426 (94.7%)
Primigravid	285 (63.3%)
Estimated gestational age, weeks	24.4 (22.7-26.2)
Slept under a bed net the previous night	226 (50.4%)
Mean hemoglobin at enrollment, g/dL	11.7 (11.6-11.9)
Participants with a history of malaria treatment during pregnancy but prior to enrollment	64 (14.2%)

### Burden of malaria during pregnancy

Including all 450 participants at enrollment and the 407 women who had at least one follow up visit, 93 episodes of malaria were detected among 79 individuals during enrollment and the antenatal follow up period. The incidence density of malaria infections was 0.6 malaria infections detected per person year of follow up. Seventy percent of women who ever had malaria were initially found to be infected at enrollment (55/79). Twenty-four women who did not have detectable parasitemia at enrollment developed malaria infection during their pregnancy and nine had malaria both at enrollment and later. Most second episodes of malaria occurred within four to eight weeks of the initial diagnosis. Among the 407 participants who had at least one follow up visit, 302 (74.2%) received three doses of SP for IPT and 82 (20.1%) received two doses.

Most infections were asymptomatic. Among all cases of peripheral parasitemia detected during the follow up period (excluding three episodes detected at delivery), 13/91 (14%) were accompanied by an objective measure of fever at the time of evaluation and 16/91 (18%) reported a history of fever. Fever detected at the visit and the report of fever in the past 48 hours were independently associated with a four-fold increase in the odds of a positive malaria smear (Odds Ratio (OR) 4.5, 95% CI 2.1-9.9 and OR 4.4, 95% CI 2.2-8.8 respectively).

### Timing of malaria infection

**Figure 1:**
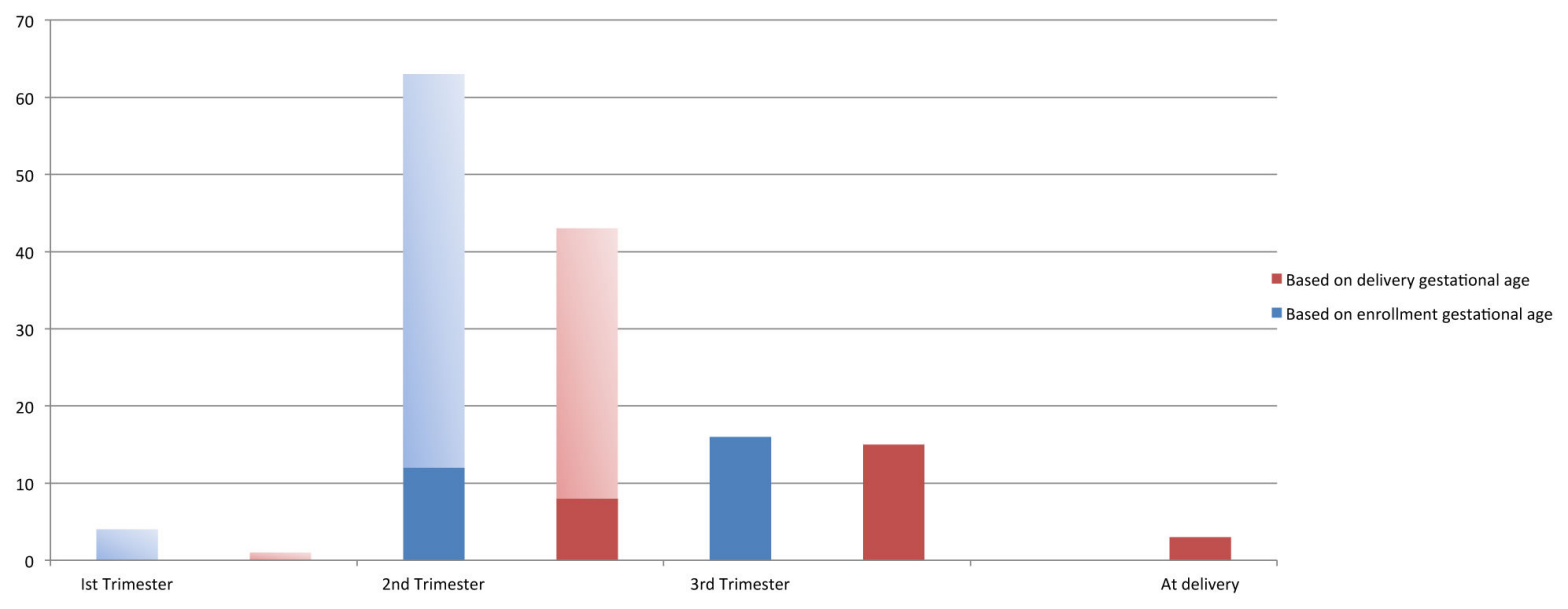
Number of participants with malaria during each trimester.

Based on last menstrual period at enrollment, four infections occurred in the first trimester, 63 in the second trimester and 16 in the third trimester. This represented 8.2%, 6.2% and 1.1% of all malaria slides obtained during those trimesters (p<0.001). After excluding the episodes of parasitemia prevalent at enrollment, the incidence of malaria was slightly higher in the second trimester than the third although the difference was not statistically significant (12/591 (2.0%) vs. 16/1398 (1.1%) of smears, p=0.1). The timing of the malaria infection based on the best assessment of gestational age at delivery had a similar distribution, but fewer infections were classified as occurring in the first trimester. Three episodes of peripheral parasitemia were detected at delivery. For two of the participants with malaria at delivery, this was their only episode of malaria and negative malaria smears were obtained within four weeks prior to delivery. For the third participant, this was a positive malaria smear four weeks after a previous episode of parasitemia that was treated with artemether-lumefantrine. The timing of the peripheral malaria infections is shown in Figure 1.

 The number of participants with malaria during each trimester based on estimated gestational age at enrollment (blue) and estimated age at delivery (red) is shown, with lighter shading indicating infections detected at enrollment.

### Placental histology

Histopathology results were available for 306 placentas from singleton deliveries. Four twin deliveries were excluded. Seventy-five (24.5%) had evidence of malaria infection. Of the women who had placental malaria, four (5.3%) had parasites detected, 62 (82.7%) had pigment detected and nine (12.0%) were found to have both parasites and pigment. Among the 60 participants known to have malaria during pregnancy and histopathology results, 47 had positive findings on histology, representing a sensitivity of 78.3% of placental histology for identifying prenatal peripheral parasitemia. There were 248 participants with available placental histopathology results and no positive malaria smears during pregnancy, 220 had no evidence of malaria on placental histology, representing 88.7% specificity. One placenta was collected from a woman who had no malaria smears obtained after enrollment. Participants whose placentas were not analyzed were younger and more likely to be in their first pregnancy than women who did have histopathological results (mean age 19.2 vs. 20.4 years and 34.7% vs. 22.4% primigravid respectively). However, the prevalence of malaria at screening and the incidence of malaria during follow up were the same.

### Risk factors for placental malaria

No demographic or socio-economic factors were associated with placental malaria, but peripheral malaria and the presence of anemia at enrollment were associated with placental malaria. Infection present at the time of enrollment in antenatal care was more strongly associated with placental infection than malaria occurring during the follow up period. There was a trend towards younger age being associated with placental malaria infection. In multivariate analysis, malaria present at enrollment, malaria during the follow up period and hemoglobin at enrollment were independently associated with placental malaria. Results of univariate and multivariate analysis are shown in Table 2.

**Table 2:** Risk factors for placental malaria.

**Risk factor**	**Without placental malaria**	**With placental malaria**	**P value univariate analysis**	**Multivariate analysis - Odds ratio (95% CI**)**^1^**
Malaria infection at screening, proportion (%)	3/230 (18.6%)	35/75 (46.7%)	<0.001	68.3 (19.0-245.7)
Any malaria during the antenatal period including participants with at least one follow up visit, proportion (%)	9/230 (3.9%)	12/75 (16.0%)	<0.001	12.6 (4.6-34.1)
Mean hemoglobin at enrollment, gm/dL	12.0	10.6	<0.001	0.7 (0.5-0.8)
Maternal age, years	20.7	19.9	0.06	0.9 (0.8-1.0)
Primigravid, proportion (%)	138/231 (59.7%)	32/75 (42.7%)	0.79	
Education beyond primary school, proportion (%)	169/231 (73.2%)	48/75 (64.0%)	0.14	
Treatment with antimalarials before enrollment, proportion (%)	36/231 (15.6%)	13/76 (17.1%)	0.85	
Slept under a bed net, proportion (%)	124/230 (53.9%)	36/75 (48.0%)	0.50	
Mean number of SP doses	2.9	2.8	0.1	

^1^ Only variables that were associated with placental malaria in univariate analysis were included in the multivariate analysis.

### Timing of malaria infection and placental pathology

The relationship between the timing of malaria infection and specific placental findings is shown in Table 3 by comparing the odds of having placental malaria with peripheral malaria infection during the second and third trimester and at delivery. Overall, infection during the second trimester was more strongly associated with evidence of placental malaria compared to malaria in the third trimester and this was due to the increased odds of hemozoin deposition in the placenta. There was a weak association between infection in the third trimester and parasites visualized in the placenta. All three women with parasites in the peripheral blood at delivery had parasites seen in their placentas.

**Table 3:** Associations between the timing of peripheral malaria infection and placental pathology.

	Peripheral malaria detected during second trimester	Peripheral malaria detected during third trimester	Peripheral infection at delivery
Proportion without placental malaria (%)	5/230 (2.1)	5/230 (2.1)	0/230 (0)
Proportion with placental malaria (%)	36/75 (48.0)	10/75 (13.3)	3/75 (4.0)
Odds ratio (95% confidence interval)	6.8 (4.0-11.2)	2.1 (1.4-3.2)	NC
Proportion without hemozoin in the placenta (%)	5/234 (21.4)	5/234 (21.4)	1/234 (0.4)
Proportion with hemozoin in the placenta (%)	36/71 (50.1)	10/71 (41.1)	2/71 (2.8)
Odds ratio (95% confidence interval)	7.4 (4.5-12.4)	2.2 (1.4-3.3)	1.8 (0.9-3.4)
Proportion without parasites in the placenta (%)	40/292 (13.7)	12/292 (4.1)	0/292 (0)
Proportion with parasites in the placenta (%)	1/13 (7.6)	3/13 (23.1)	3/13 (23.1)
Odds ratio (95% confidence interval)	0.8 (0.3-2.2)	1.8 (1.0-3.1)	NC

^NC: Not calculated^

### Maternal and infant outcomes

Information on maternal hemoglobin levels at delivery was available for 321 women. The mean hemoglobin level was 12.8 g/dL (95% CI 12.6-13.0). Thirty-five women had anemia at delivery with four (1.3%) women having moderate and 31 (9.7%) having mild anemia. Malaria during pregnancy overall and placental malaria were not associated with differences in maternal hemoglobin. Peripheral malaria at the time of delivery was associated with increased rates of maternal anemia compared to infections during other trimesters (OR 2.4, 95% CI 1.2-4.6, p=0.01).

Birth outcomes were available for 330 newborns. Among nine women (2.4%) whose deliveries resulted in stillbirths, eight never had malaria detected during pregnancy or in the placenta whereas one had four episodes of malaria with pigment detected in the placental specimen. Thirteen neonatal deaths occurred. Eleven of the deaths occurred in infants whose mothers did not have any evidence of peripheral or placental malaria during pregnancy One infant was born to a mother who had malaria during the second trimester but whose placenta did not show any infection and another infant was born to a mother who did not have malaria detected during pregnancy but did have malaria pigment found in the placenta.

The mean birth weight was 2.8 kilograms (95% CI 2.8-2.9) and the estimated gestational age at delivery was 38.6 weeks (95% CI 38.2-38.9). Fifty (15.1%) infants were born preterm, 58 (17.9%) were low birth weight and 43 (13.3%) were small for gestational age. Birth weight and gestational age were not associated with exposure to malaria during pregnancy overall or in any one trimester except for the finding that mothers with malaria at delivery were at a significantly higher risk of delivering a small for gestational age infant than mothers with malaria at other times during pregnancy (2/3 infants born to mothers with peripheral malaria at delivery were small for gestational age, OR 2.2, 95% C 1.1-2.4, p=0.03). Number of episodes of malaria during pregnancy was also not associated with any of the adverse infant outcomes.

## Discussion

In this study the timing of malaria during pregnancy had significant effects on the clinical outcomes of pregnancy. Infection that occurred early in pregnancy, before or just at the onset of the initiation of antenatal care was strongly associated with evidence of placental infection. Lower hemoglobin at first antenatal visit, even in the absence of parasitemia, was a strong predictor of placental malaria. In the context of SP IPTp and also active case detection and treatment, malaria during pregnancy and placental malaria were not associated with adverse maternal or infant outcomes with the exception of the association of peripheral malaria infection found at delivery with small for gestational age infants and maternal anemia.

Previous studies have not provided a consistent characterization of the effects of malaria during different periods of gestation on maternal and infant outcomes. A similar study conducted in Malawi, but with two rather than three doses of SP IPT, demonstrated that the number of episodes of malaria during pregnancy was associated with maternal anemia, and malaria during the second trimester was associated with low birth weight [5]. In Benin, malaria infections detected at the first antenatal visit were associated with low birth weight and anemia at delivery while late infection was associated with only maternal anemia [6]. In that study, women were given two doses of SP during pregnancy and malaria infections detected by rapid diagnostic test were treated when women were symptomatic. In another study in Burkina Faso, malaria during the first trimester was significantly associated with low birth weight [7]. Participants were identified through regular home visits for pregnancy screening and as a result the surveillance was able to capture malaria episodes very early in pregnancy. The differences among the previous studies likely reflect differences in study design, including the type of screening, rigor of case detection and method for assessing gestational age, as well as the local malaria transmission dynamics and application of control measures.

The results of this study confirm the moderate usefulness of monitoring placental malaria as a proxy for either peripheral malaria infection during pregnancy or as a surrogate for adverse outcomes associated with pregnancy malaria. The specific histological findings did generally correlate with the expected findings that pigment represents prior infection and parasites represent recent or on-going active infection [8]. However, this study confirms that placental histology does not capture all documented cases of prenatal infection.

The prevalence of malaria was highest at the first visit for antenatal care and was infrequently detected during follow up. This points to the continued ability of SP to suppress microscopically detectable blood stage infection. Because there was no untreated control arm for this study, it is not possible to measure the effect of SP IPTp on placental infection. However, the high rate of placental abnormalities associated with malaria infection, particularly pigment deposition, suggests that even three doses of SP IPTp do not provide adequate protection against or cure placental infection. This may be due to poor curative efficacy of SP against malaria in Malawi, like other parts of eastern Africa. Another contributor might be the late timing of the initial SP dose. Women who present with malaria at the initial antenatal visit are at the highest risk of placental infection. The intervention may be administered after parasites have already sequestered in the placenta.

Although active peripheral malaria infection at delivery was associated with low birth weight and small for gestational age infants, it is not biologically plausible that a new infection that late in pregnancy would have a significant impact on birth weight. Rather, we suspect that patent parasitemia at delivery may reflect on-going low density parasitemia that was likely occurring throughout pregnancy and led to reduced birth weight. This finding was a result of a small number of cases so further investigation is required to confirm and explore this association.

Several features of the study design limit the generalizability of this study. Most women were enrolled late in the second trimester of pregnancy, so we had limited information about the effects of malaria infection during the first half of the pregnancy when placental implantation and transformation of the maternal circulation occurs. Detection and treatment of malaria in the absence of symptoms is not currently standard practice in most malaria endemic countries. However, increasing the attention to the role of asymptomatic infection in adverse outcomes, the pervasiveness of SP resistance and the widespread availability of rapid diagnostic tests may support the addition of this strategy to public health practice. This analysis is limited to the detection of parasites detected through microscopy. Though submicroscopic parasitemia has been shown to be associated with adverse outcomes [9,10], we limited this report to results that can be ascertained during the course of routine clinical care in Malawi. We also used our best estimate of gestational age to assess the timing of the infection, using menstrual dates, fundal height and postnatal Ballard examination. Ultrasound is a more precise method to date pregnancy, though it is not routinely available in low resource settings. Finally, we had incomplete follow up data on approximately one quarter of pregnancies. Although the women who did not have their placentas collected, often because they delivered outside the catchment area, were younger than women whose placentas were examined, the burden of malaria infection was likely similar. Travel outside the city is a frequent occurrence in our highly mobile urban population. The most common reasons for loss to follow up, migration out of the study area and withdrawal of consent due to pressure from an influential family member, are unlikely to be associated with malaria outcomes.

The results of this study suggest several key public health implications. First, currently recommended interventions to prevent malaria during pregnancy may be provided too late in pregnancy to achieve the desired impacts. Presentation for antenatal care half way through pregnancy is typical throughout sub-Saharan Africa [11]. However, even in this urban setting, where malaria transmission may be lower than in rural areas, one out of every eight pregnant women had malaria infection at densities high enough to be detectable by microscopy, suggesting that even more women are infected at a submicroscopic level. Currently implemented interventions including expanding bed net coverage to all household members and ensuring access to accurate diagnosis and effective treatment for symptomatic malaria will likely lead to a decrease in infection in all populations, regardless of whether women present for timely antenatal care. Specific interventions to prevent pregnancy-associated malaria either need to occur in conjunction with efforts to encourage early attendance for antenatal care or will need to target all women of child-bearing age to have maximal impact.

The association between anemia and placental malaria suggests that screening for anemia at the first antenatal visit may help to identify pregnant women at risk for placental infection. Anemia may be a marker for infections sequestered in the placenta that are not microscopically detectable in the peripheral blood or they may indicate an individual who is frequently infected with malaria. Hemoglobin concentration can be measured from the same finger prick used to diagnose malaria using inexpensive, field-friendly techniques. Further investigations could explore the utility of providing first line antimalarial treatment instead of or in addition to the first dose of SP IPTp.

In addition, this study supports the added benefit of screening for malaria infection even in the context of the appropriate administration of SP IPTp. We and others have demonstrated that the ability to detect and treat microscopically detectable malaria during pregnancy prevents the typical detrimental effects of pregnancy-associated malaria [5,12]. The use of antigen-based rapid diagnostic tests may further improve the protection offered by adding routine screening to IPTp. The extent to which detection and treatment of parasite antigenemia, which may entail a much higher number of treatment doses compared to restricting treatment to microscopically detectable cases, will improve outcomes is an area of active research.

## Conclusion

Malaria infection occurs most frequently early in pregnancy, before women typically seek obstetric care, pointing to the urgent need to expand antimalarial interventions outside of the antenatal clinic. The combination of three doses of SP IPTp, routine screening and treating all detectable malaria infections does not prevent placental infection but appears to mitigate the adverse clinical outcomes typically associated with placental malaria.
